# A nomogram for determining the disease-specific survival in invasive lobular carcinoma of the breast: A population study

**DOI:** 10.1097/MD.0000000000022807

**Published:** 2020-10-23

**Authors:** Rong Fu, Jin Yang, Hui Wang, Lin Li, Yuzhi Kang, Rahel Elishilia Kaaya, ShengPeng Wang, Jun Lyu

**Affiliations:** a Department of Clinical Research, The First Affiliated Hospital of Jinan University, Guangzhou, Guangdong Province; b School of Public Health, Xi’an Jiaotong University Health Science Center; c Shaanxi Cancer Hospital; d Cardiovascular Research Center, School of Basic Medical Sciences, Xi’an Jiaotong University Health Science Center; e Key Laboratory of Environment and Genes Related to Diseases of Ministry of Education, Xi’an Jiaotong University Health Science Center, Xi’an, Shaanxi, China.

**Keywords:** invasive lobular carcinoma, nomogram, prognosis, survival

## Abstract

We aimed to establish and validate a nomogram for predicting the disease-specific survival of invasive lobular carcinoma (ILC) patients.

The Surveillance, Epidemiology, and End Results program database was used to identify ILC from 2010 to 2015, in which the data was extracted from 18 registries in the US. Multivariate Cox regression analysis was performed to identify independent prognostic factors and a nomogram was constructed to predict the 3-year and 5-year survival rates of ILC patients based on Cox regression. Predictive values were compared between the new model and the American Joint Committee on Cancer staging system using the concordance index, calibration plots, integrated discrimination improvement, net reclassification improvement, and decision-curve analyses.

In total, 4155 patients were identified. After multivariate Cox regression analysis, nomogram was established based on a new model containing the predictive variables of age, the primary tumor site, histology grade, American Joint Committee on Cancer TNM (tumor node metastasis) stages II, III, and IV, breast cancer subtype, therapy modality (surgery and chemotherapy). The concordance index for the training and validation cohorts were higher for the new model (0.781 and 0.832, respectively) than for the old model (0.733 and 0.779). The new model had good performance in the calibration plots. Net reclassification improvement and integrated discrimination improvement were also improved. Finally, decision-curve analyses demonstrated that the nomogram was clinically useful.

We have developed a reliable nomogram for determining the prognosis and treatment outcomes of ILC. The new model facilitates the choosing of superior medical examinations and the optimizing of therapeutic regimens with cooperation among oncologists.

## 1 Introduction

Breast cancer is a heterogeneous disease with multiple prognoses.^[1]^ Invasive lobular carcinoma (ILC) is the most-common specific type of breast cancer, which accounts for 15% of all cases and presents with a distinct morphology and clinical behavior compared with invasive carcinoma of no special type.^[2]^ ILC has unique clinical, pathological, and radiographic features that suggest it is a distinct clinical entity.^[3]^ Over the last 2 decades ILC has accounted for 25,000 to 30,000 new cases of breast cancer in the USA annually, and its incidence is increasing, especially among postmenopausal women. If considered an independent cancer type, ILC would be the sixth-most-common cancer in women, with an occurrence frequency similar to those of non-Hodgkin's lymphoma and melanoma.^[4,5]^ ILC tumors typically have a good prognosis, low histology grade, and positivity for the estrogen receptor; however, they can be strongly metastatic and are the main cause of cancer deaths among women in many countries worldwide.^[2,6]^ There is increasing evidence that ILC is clinically unique, and that its early diagnosis and prognosis are especially important.

The American Joint Committee on Cancer (AJCC) staging system has been widely used to determine clinical treatment strategies and assess clinical risks. However, there are limitations in using the AJCC staging system alone to predict the prognosis of patients, and the overall outcomes can vary widely for tumors at the same stage. The clinical uniqueness of ILC means that novel prognostic tools are needed to increase the accuracy of predicting the survival of affected patients.^[7]^

A nomogram is a convenient diagrammatic representation of a mathematical model that combines various important factors to predict a specific endpoint.^[8]^ A nomogram can therefore be an effective visual tool for improving the predictive accuracy of the prognosis in individual patients and for providing individualized prognostic information based on a combination of parameters.^[9–11]^ Nomograms have been found to be helpful for clinicians making decisions and predicting the outcome of an individual, thereby bringing benefits to both clinicians and patients.^[12]^ The aim of this study was to establish a comprehensive prognostic evaluation system for ILC patients and validate its predictive accuracy.

## 2 Meterials and methods

### 2.1 Data source

Patient information was collected from the Surveillance, Epidemiology, and End Results (SEER) database, which covers approximately 30% of the population of the USA and includes cases from 18 registries. Informed patient consent is unnecessary when utilizing data from the SEER program that does not include personal identifying information. We searched for ILC patients using the ICD-O-3 (third revision of the International Classification of Diseases for Oncology) histological subtype code 8520/3. We used the sixth edition of the AJCC staging system and restricted our search to between 2010 and 2015.

### 2.2 Variable selection

The analyzed demographic variables of the patients included age at diagnosis, race, sex, marital status, primary tumor site, histology grade, laterality, AJCC tumor node metastasis (TNM) stage, AJCC T stage, AJCC N stage, AJCC M stage, treatment status (surgery, radiation, and chemotherapy), bone metastasis, and breast cancer subtype.

Age was classified into <40, 40 to 59, 60 to 79, and ^3^80 years. Race was classified into white, black, and other. Sex was classified into female and male. Marital status was classified into married, unmarried, and unknown. The primary tumor site was classified into the axillary tail, central portion, lower-inner quadrant, lower-outer quadrant, upper-inner quadrant, and upper-outer quadrant of the breast. The histology grade was classified into grades I, II, and III. Laterality was classified into left primary origin, right primary origin, and only 1 side. The AJCC TNM stage was classified into stages II, III and IV. The AJCC T stage was classified into stages T1, T2, T3, and T4. The AJCC N stage was classified into stages N1, N2, and N3. The AJCC M stage was classified into stages M0 and M1. Surgery, radiation, and chemotherapy were classified into receiving and not receiving/unknown. Bone metastasis was classified into yes and no/unknown. The breast cancer subtype was classified into luminal A, luminal B, HER2 enriched, and triple negative. Patients with missing or unknown survival time were excluded.

### 2.3 Statistical analysis

Continuous variables that conformed to a normal distribution were expressed as mean ± SD values, while categorical variables were expressed as frequencies and percentages. Multivariate Cox proportional-hazards regression models were applied to determine the factors associated with survival. Based on the predictive model with the identified prognostic factors, a nomogram was constructed for predicting the 3-year and 5-year survival rates of ILC patients.

The nomogram was tested by measuring discrimination and calibration curves in both a training cohort (internally) and a validation cohort (externally). The predictive accuracy of the nomogram was evaluated using the concordance index (C-index) and the area under the time-dependent receiver operating characteristic curve (AUC). The C-index quantified the predictive ability of the model, and ranged from 0.5 to 1.0. Calibration plotting was used to evaluate the agreement between the predicted probabilities and the actual outcomes. Bootstrapping with 500 resamples was used to evaluate both discrimination and calibration. The relative integrated discrimination improvement (IDI) and the net reclassification improvement (NRI) were calculated to estimate the accuracy of the model in predicting outcomes with and without the application of prognostic therapies. Decision-curve analyses (DCAs) were used to assess the clinical value of the predictive models. All statistical analyses were performed using SPSS (version 25.0, SPSS, Chicago, IL) and R software (version 3.6.1), with a 2-sided probability value of *P* < .05 considered to be indicative of statistical significance.

### 2.4 Ethical review

Because of cancer is a reportable disease in every state of the USA, informed patient consent is not required. Once the data use agreement is signed, data on cancer research is freely available to the public.

## 3 Results

### 3.1 Baseline characteristics

There were 4155 eligible ILC patients identified in the SEER database. For nomogram construction and validation, we randomly assigned 70% of the patients to the training cohort (n = 2908) and 30% to the validation cohort (n = 1247). The survival period was known for all of the patients included in this study. The largest proportions of the patients were aged 60 to 79 years (49.1% and 49.2% in the training and validation cohorts, respectively), white (83.5% and 83.1%), female (both 99.9%), married (82.6% and 80.0%), and had a primary tumor site in the upper-outer quadrant of the breast (57.2% and 57.3%). Many patients were histology grade II, AJCC TNM stage II, AJCC stage T2, AJCC stage N1, AJCC stage M0, and luminal-A breast cancer subtype. About 94% of patients had undergone surgery. More than half of the patients had received both radiation and chemotherapy. Table 1 lists the clinicopathological characteristics of all of the included patients.

**Table 1:**
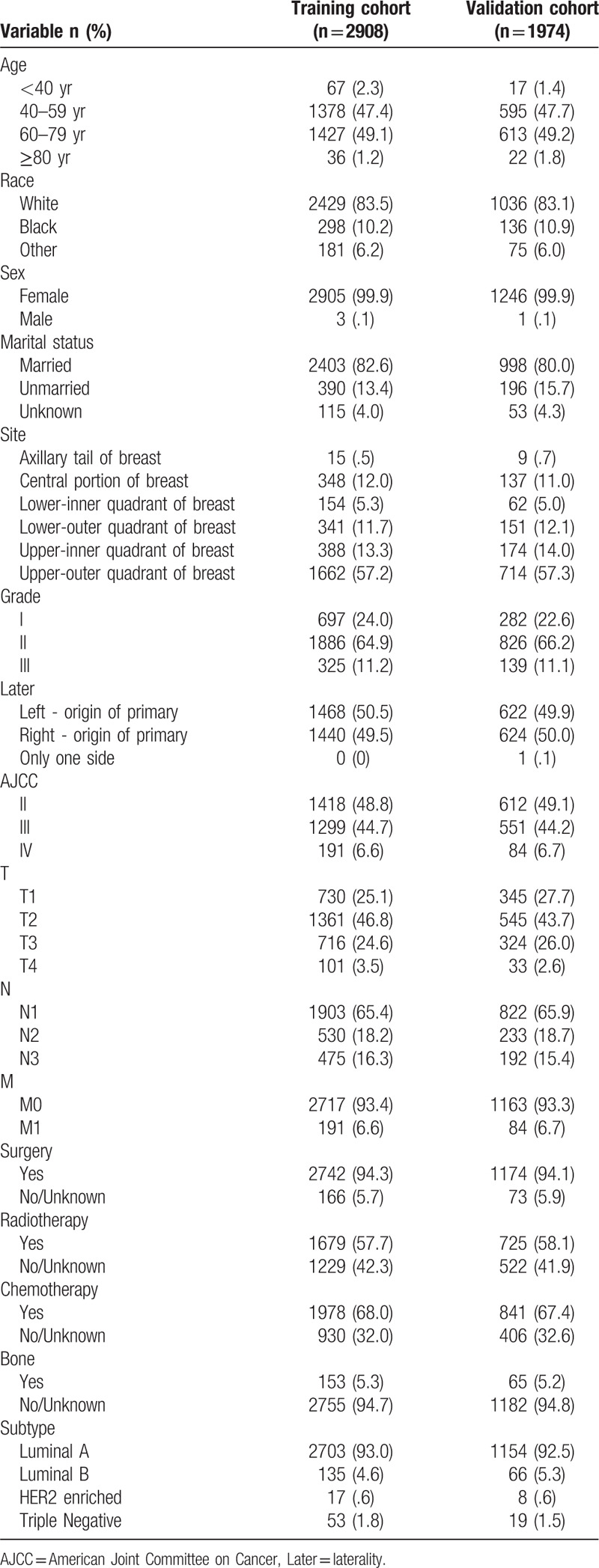
Patient characteristics in the study.

### 3.2 Multivariate cox regression analysis

Several independent prognostic variables were identified by analyzing the multivariate model. Data on the age at diagnosis, primary tumor site, histology grade, AJCC stage, surgery status, chemotherapy status, and breast cancer subtype were entered into multivariate Cox regression analyses, which demonstrated that AJCC TNM stage III (hazard ratio [HR] = 3.280 vs AJCC TNM stage II, *P* < .001), AJCC TNM stage IV (HR = 12.932 vs AJCC TNM stage II, *P* < .001), no surgery (HR = 2.338, *P* < .001), no chemotherapy (HR = 1.435, *P* < .05), and triple-negative breast cancer subtype were independent risk factors for the survival of ILC patients (Table 2).

**Table 2:**
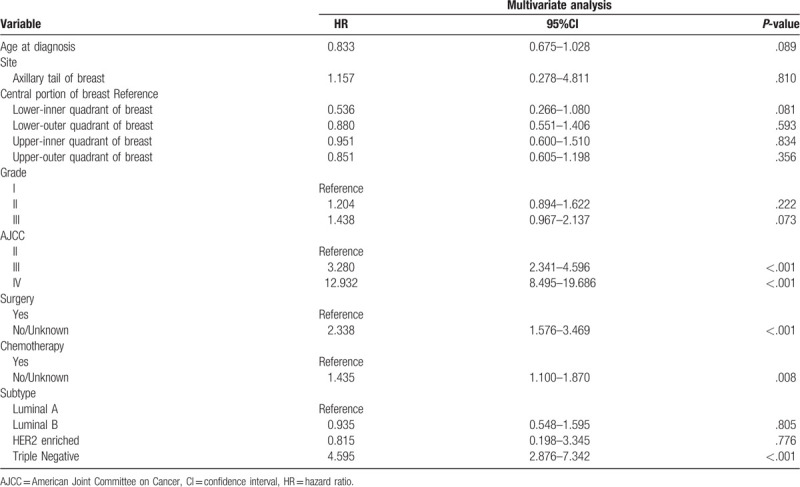
Selected variables by multivariate Cox regression analysis in the training cohort.

### 3.3 Nomogram construction

**Figure 1:**
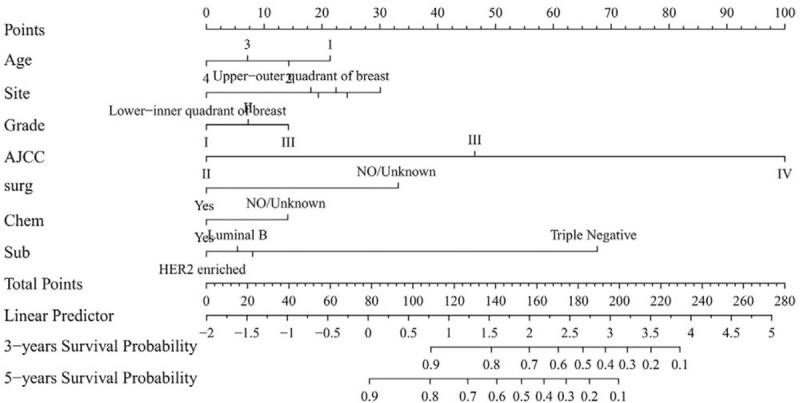
Nomogram predicting 3- and 5-year survival, AJCC = area under the time-dependent receiver operating characteristic curve, Chem = chemotherapy, Sub = breast cancer subtype, surg = surgery.

The data from the logistic regression model were used to construct a nomogram. Each variable included in the nomogram was assigned a value related to the degree to which it influenced the outcome variable in the model. Each predictive factor was scored according to a set scale. The total summation score (in points) on this nomogram was then converted into the probabilities of 3-year and 5-year survival. The nomogram showed that the AJCC stage was the most important contributor to the prognosis, followed by the breast cancer subtype, surgery status, primary tumor site, age, histology grade, and chemotherapy status (Fig. 1).

### 3.4 Performance of the nomogram

**Figure 2:**
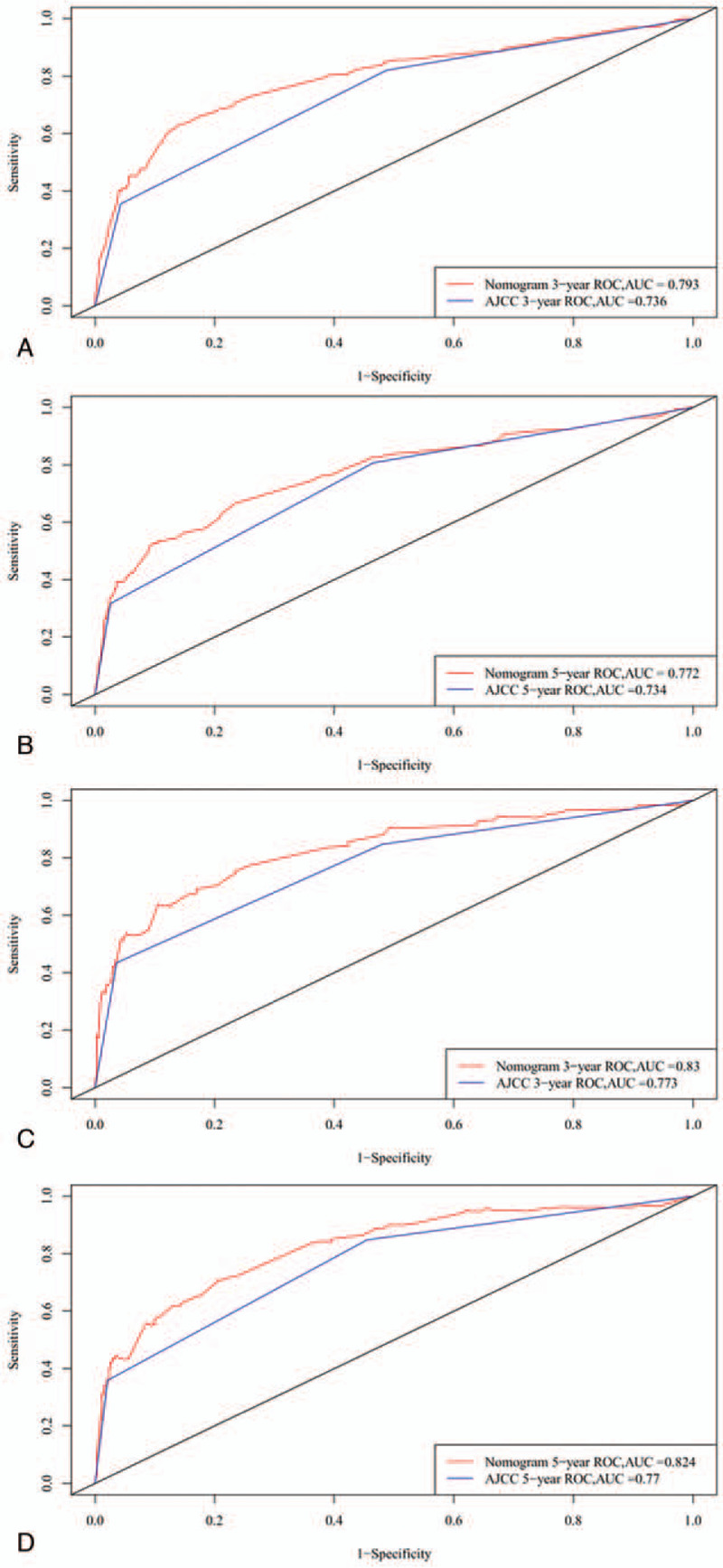
ROC curves. The ability of the model to be measured by the C index. A and B came from the training set, and C and D came from the validation set. ROC = receiver operating characteristic.

**Figure 3:**
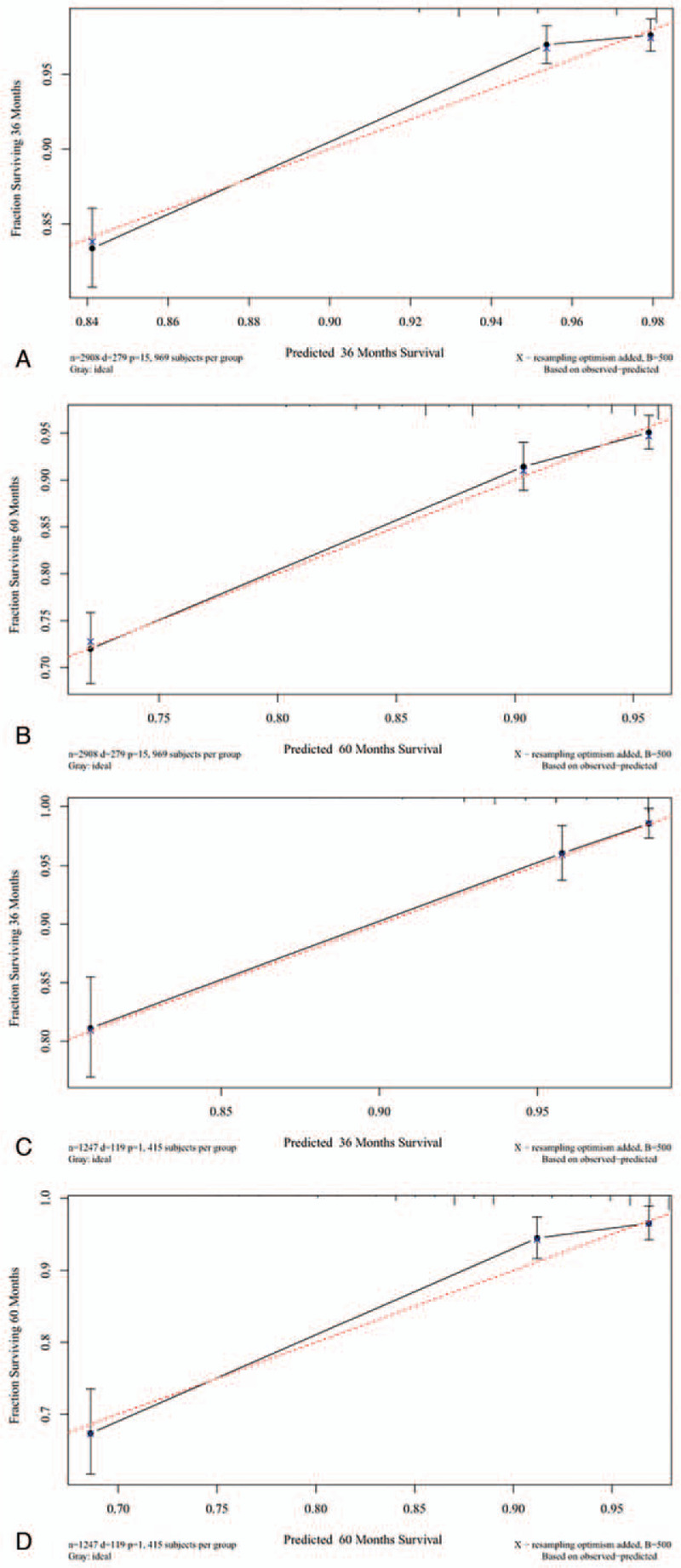
Calibration plots. Show the relationship between the predicted probabilities base on the nomogram and actual values of the train set (A and B) and validation set (C and D).

The C-indexes were higher for the nomogram (0.781 and 0.832 for the cohort and validation cohorts, respectively) than for the AJCC staging system (0.733 and 0.779). For the nomogram, the AUCs of the training cohort (0.793 at 3 years and 0.772 at 5 years) and validation cohort (0.83 and 0.824, respectively) indicated that the model had better discriminative ability than the 6 edition of the AJCC staging system (Fig. 2). Calibration plots of the nomogram showed that the predicted 3-year and 5-year survival probabilities for the training and validation cohorts were almost identical to the actual observations (Fig. 3).

### 3.5 Validation of the nomogram

The NRI values were 0.202 (95% confidence interval [CI] = 0.119 to 0.332) and 0.117 (95% CI = 0.074–0.278) for 3 years and 5 years of follow-up examinations, respectively, in the training cohort; the corresponding values in the validation cohort were 0.269 (95% CI = 0.110 to 0.446) and 0.180 (95% CI = –0.024 to 0.345). These values indicate that the nomogram represented a great improvement. Similarly, the IDI values for 3 years and 5 years of follow-up were 0.028 and 0.030 in the training cohort, respectively, and 0.040 and 0.033 in the validation cohort, which indicate that the new model had superior predictive performance.

### 3.6 Decision-curve analysis

The results of the DCA graphically showed that the new model yielded greater net benefits for the 3-year and 5-year survival than the traditional AJCC staging system, which indicates that the model is clinical useful and can play a useful practical role in decision-making.

## 4 Discussion

ILC, which is also known as infiltrating lobular carcinoma, is the second-most-common histological type of breast cancer, and its incidence is increasing.^[13]^ Although ILC is less common than invasive ductal breast cancer (IDC), the proportion of cases with ILC is gradually increasing.^[14,15]^ ILC tumors have a better long-term outlook and tend to be less aggressive than IDC tumors, but they are inclined to metastasize to the genital tracts, the gastrointestinal system, and meninges, and additional more commonly transfer to atypical sites.^[16–20]^ This situation indicates the need for further research into the prognosis of ILC. The early identification of high-risk ILC patients is helpful for providing adjuvant treatment or trials. Although the existing clinical AJCC staging system provides meaningful predictions of the prognosis of ILC patients, it has limitations in estimating the clinical risk of ILC. We have therefore developed a comprehensive predictive model that includes not only the patient demographics but also therapies and other clinical parameters. Our novel model can provide an independent data set for ensuring fairer model assessments.

This study was based on the large-sample database of the SEER program, which started with 8 registries in 1973 and has continuously increased with the addition of other participating sites over time. Currently the database includes 18 geographically diverse areas representing 30% of the USA population, with efforts made to accurately reflect the racial, economic, and social diversity of the country.^[21–23]^ In order to obtain reliable research results, we identified 4155 patients with ILC from 2010 to 2015 in the SEER database.

Table 1 indicates that most of the patients in our study tended to be older, white, female, and married, had a primary tumor site in the upper-outer quadrant of the breast, and had received treatment with surgery, radiotherapy, and chemotherapy. ILC patients are more likely to have hormone-receptor-positive tumors, which are typically of a lower histology grade, and these results are consistent with previous research findings.^[24–27]^ The results of the multivariate Cox regression presented in Table 2 indicate that surgery and chemotherapy were protective factors. As many studies have shown, selecting surgical treatment for ILC patients is an appropriate and acceptable option and offers superior local control.^[28,29]^ Although ILC often responds poorly or not at all to chemotherapy, there is currently insufficient evidence to support these assumptions.^[16,17,30]^ Moreover, ILC patients exhibit better disease-free survival and overall survival, especially among those at a high risk.^[3,31]^ Similarly, from our nomogram (Fig. 1) it is evident that the prognosis is worse for patients who do not receive chemotherapy, while the probabilities of residual disease and local recurrence decrease after chemotherapy.^[32]^ Although the luminal-B and HER2-enriched breast cancer subtypes accounted for only a small proportion of the tumors in the present study, they exhibited significant responses to chemotherapy.^[31,33]^ Therefore, in view of these objective results, surgery and chemotherapy can be considered to be protective factors for ILC patients.

A nomogram is a graphical representation of a complex statistical formula that includes multiple variables and provides an easy-to-understand answer to a focused question. In this study we developed and validated an easy-to-use nomogram for predicting the 3-year and 5-year survival rates in ILC patients. Our new nomogram model contains a large number of risk factors that are easily collectable from historical records. The nomogram was able to identify a high-risk subgroup of patients who might need intensive therapy. To the best of our knowledge, our nomogram is the first for predicting the 3-year and 5-year survival rates, and we evaluated the performance of the model by using C-indexes, calibration, NRI, IDI, and DCA. This study generated receiver operating characteristic curves to compare the performances of the new nomogram and the traditional AJCC staging system based on AUCs.

**Figure 4:**
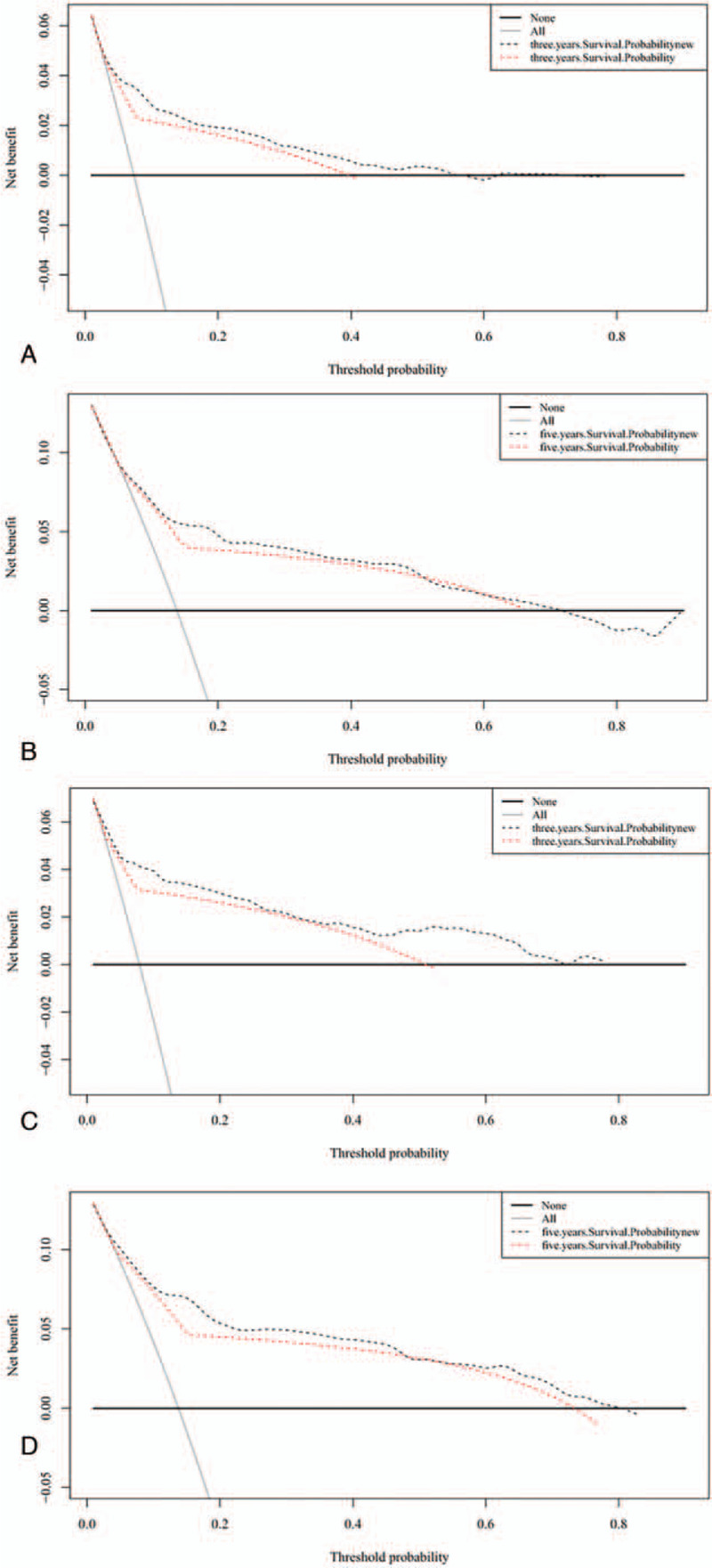
Decision curve analysis. In the figure, the abscissa is the threshold probability, the ordinate is the net benefit rate. The horizontal one indicates that all samples are negative, and all are not treated, with the benefit of 0. The oblique one indicates that all samples are positive. The net benefit is a backslash with a negative slope. A and B came from the training set, C and D came from the validation set.

The AUC was larger for the nomogram than for the AJCC staging system alone. Our nomogram also showed good discrimination, with C-indexes of 0.781 and 0.832 for the training and validation cohorts, respectively, which are higher than the values for the AJCC staging system. These results indicate that our nomogram model provided a good fit to the randomly allocated training and validation cohorts. To further confirm the good performance of our novel model, we used calibration curves to depict the calibration according to the consistency between the predicted probabilities and observed outcomes. Figure 3 shows that the nomogram predictions were well calibrated. We also applied IDI and NRI to evaluate the performance of our survival model, with the positive results further demonstrating the superior performance of the nomogram. Figure 4 shows the results of DCAs, with the abscissa corresponding to the threshold probability and the ordinate being the net benefit rate.^[34–36]^ The figure illustrates that the new model yielded net benefits that were superior to those of the traditional AJCC staging system. These results together demonstrate that our nomogram would provide useful information about the risks and benefits of certain treatment plans, thereby helping clinicians to make good decisions and even provide psychological support.

## 5 Limitations

This study analyzed a large population from the high-quality SEER database, but the utilization of retrospective data would have introduced unavoidable bias. Second, information was not available for some of the cases, and we only included patients for whom complete information was available, which would have excluded many patients and hence introduced selection bias. Finally, the predicted values calculated by using the nomogram only represent reference information that should be interpreted by clinicians, rather than absolutely accurate prognoses.

## 6 Conclusion

In summary, nomograms are an important component of modern medical decision-making. We have developed and validated a highly accurate ILC-prognosis nomogram based on the SEER database. Future studies are needed to externally validate the nomogram.

## Acknowledgment

The authors acknowledge the efforts of the Surveillance, Epidemiology, and End Results (SEER) program registries for creating the SEER database.

## Author contributions

**Conceptualization**: Rong Fu, Jun Lyu.

**Data Curation**: Jin Yang, ShengPeng Wang, Jun Lyu.

**Methodology**: Hui Wang, Yuzhi Kang, Li Lin, ShengPeng Wang, Jun Lyu.

**Software**: Jin Yang, Rahel Elishilia Kaaya, Jun Lyu.

**Writing – original draft**: Rong Fu.

**Writing – review & editing**: ShengPeng Wang, Jun Lyu.
